# Asymmetric Dimethylarginine Levels are Highly Associated With Atrial Fibrillation in an Elderly Population

**DOI:** 10.4021/cr175w

**Published:** 2012-05-20

**Authors:** Ingebjorg Seljeflot, Sara R. Ulimoen, Steve Enger, Vibeke Bratseth, Harald Arnesen, Arnljot Tveit

**Affiliations:** a Center for Clinical Heart Research, Oslo University Hospital Ulleval, Norway; b Department of Cardiology, Oslo University Hospital Ulleval, Norway; c Faculty of Medicine, University of Oslo, Norway; d Medical Research Department, Department of Medicine, Vestre Viken Hospital Trust, Rud, Norway

**Keywords:** Atrial fibrillation, Elderly, Endothelial dysfunction, ADMA, Von Willebrand factor, Endothelial cell adhesion molecules

## Abstract

**Background:**

The importance of endothelial dysfunction in atrial fibrillation (AF) is not clarified. The aim of this study was to evaluate endothelial dysfunction assessed by selected inflammatory and haemostatic endothelial markers and nitric oxide (NO) associated variables as related to the presence of AF in an elderly population. NO is known to express anti-thrombotic as well as vasoactive properties.

**Methods:**

This is a cross sectional study of 75-year old subjects with AF (n = 62) and control subjects in sinus rhythm (n = 124), matched for gender. Fasting blood samples were collected for analyses of asymmetric dimethylarginine (ADMA), an endogenous inhibitor of NO-synthase, L-arginine, E-selectin, vascular cell adhesion molecule-1 (VCAM-1) and von Willebrand factor (vWF).

**Results:**

Levels of vWF and ADMA were significantly higher in AF patients vs controls (P = 0.023 and P < 0.001, respectively) and the L-arginine/ADMA ratios were lower (P = 0.015), the latters still significant after adjustment for relevant covariates (P = 0.007 and P = 0.037, respectively). No significant differences in the levels of VCAM-1 and E-selectin were observed between the groups. When dividing the ADMA levels into quartiles there was a significant trend for having AF with increasing levels of ADMA (P < 0.001) with a cut-off at the 25th percentile (< 0.54 µmol/L), giving an adjusted OR for having AF of 12.46 (95% CI 3.11 - 49.86) (P < 0.001) with higher levels. A similar inverse trend was seen for the L-arginine/ADMA ratio.

**Conclusion:**

Our population of 75-year-old AF patients had significantly impaired endothelial function assessed by increased levels of vWF, and more pronounced by high levels of ADMA. The results indicate AF in the elderly to be closely associated with the regulatory pathway of NO.

## Introduction

The prevalence of atrial fibrillation (AF) is high in the elderly and increases with increasing age [1]. Age seems to add to other independent risk factors for AF, like gender, prior AF, hypertension, chronic renal insufficiency, obesity and inflammation [1-3]. AF is associated with an increased risk of thromboembolisms, and lately, AF has also been shown associated with endothelial dysfunction [4]. However, the underlying pathophysiological mechanisms in AF have not been fully elucidated. It has been discussed along with thrombogenesis and the proinflammatory state in AF [5-7] both closely related to endothelial dysfunction.

Different non-invasive methods for evaluation of endothelial dysfunction in AF have been used, including measurement of circulating endothelium related biomarkers, like von Willebrand factor (vWF) and soluble endothelial cell adhesion molecules (i.e intercellular adhesion molecule-1, vascular cell adhesion molecule-1 (VCAM-1) and E-selectin). These biomarkers have to some extent been studied in relation to AF [8].

Central to endothelial function is the production of endothelial nitric oxide (NO), an important regulator of vascular tone and haemostatic balance. Impaired vasodilation is suggested to be caused by inhibition of NO generation by the amino acid asymmetric dimethylarginine (ADMA), an important endogenous inhibitor of NO synthase (eNOS) [9-10]. ADMA is formed by methylation of proteins containing L-arginine, which is the substrate of NOS. Several studies support the view that the ratio between L-arginine and ADMA is important for the regulation of eNOS activity [11-12].

Increased levels of ADMA have been reported in different disease states with high risk for AF, like coronary artery disease, obesity, hypertension and chronic heart failure [13-16]. There is, however, limited data on the importance of ADMA and L-arginine in AF. It was reported that ADMA levels in patients with acute AF were significantly increased when compared to patients with chronic AF and in healthy controls [17]. Results from another study indicate that high levels of ADMA are associated with an increased risk of AF recurrence within 1 month after electrical cardioversion [18]. The latter could not be confirmed in our recently published study in which neither L-arginine, nor ADMA were found to be predictive of rhythm outcome after cardioversion [19].

The aim of the present study was to evaluate whether AF was related to endothelial dysfunction assessed by the levels of ADMA and L-arginine, as well as levels of inflammatory and haemostatic endothelial markers (E-selectin, VCAM-1 and vWF) in a population based cross sectional study in elderly individuals.

## Material and Methods

### Study design and population

This study was a substudy of the Asker and Baerum Atrial Fibrillation study [20]. Briefly, all residents in Asker and Baerum counties in eastern Norway born in the year 1930 were invited to participate in the study. The study was performed at the county hospital, which is the only hospital in this area. Out of 1,117 invited subjects, 916 were finally included. AF was found present in 92 subjects (10.0%), including subjects with persistent/permanent AF.

All subjects with AF were invited to participate in this substudy. In addition, a control group in sinus rhythm twice that size was included. To assure equal gender distribution, for each subject with AF identified, the next 2 subjects in sinus rhythm of the same gender were invited to participate. The study was approved by the Regional Ethics Committee, and all participants provided written, informed consent in accordance with the revised Declaration of Helsinki before enrolment.

Most subjects were examined in the outpatient clinic, and home visits were arranged for those unable to go to the hospital. Twelve-lead electrocardiograms (ECG) were recorded in the supine position after five minutes rest (10-second printouts with 50 mm/s and 10 mm/mV on a Schiller AT-101 (Baar, Switzerland)). ECG recordings were analyzed automatically and reviewed by a specially trained study nurse. Abnormal findings were reviewed by an experienced internist. Blood pressure was measured in the supine position after 10 minutes rest. If the initial blood pressure was higher than 160/95, a repeated measure was performed, and the lowest measurement was registered. Hypertension was defined as systolic blood pressure > 160 mmHg and/or diastolic blood pressure > 95 mmHg, or current use of any antihypertensive medication. Heart failure was defined as a diagnosis of heart failure in the hospital records or based on information provided by the patient’s primary physician if diagnosed elsewhere. The diagnosis was based on echocardiographic findings (most cases) and/or clinical or radiological signs of congestive heart failure. Similarly, coronary heart disease was defined based on previously diagnosed myocardial infarction, typical symptoms and a positive stress test, scintigraphic examination or coronary angiography.

Medical history and current medication were recorded from questionnaires and by interview. Supplementary information was retrieved from hospital and general practitioners records. All study procedures were performed between September 2004 and September 2005.

### Laboratory methods

Venous blood samples were collected from all subjects in fasting condition after 10 minutes rest. Routine clinical chemistry analyses were performed by use of conventional methods. Additionally, serum was prepared within 1 hour by centrifugation at 2000 x g for 10 - 15 minutes in room temperature. Blood samples for citrated and EDTA plasma were stored on ice until centrifugation at 2500 x g for 15 - 20 minutes at 4 °C. All samples were frozen at -70 - 80 °C until analysed.

Serum was used for determination of cell adhesion molecules, and citrated plasma was used for vWF, all analyzed by commercial ELISA methods (R & D Systems Europe, Abingdon UK and Asserachrom vWF, Stago Diagnostica, Ansiere, France, respectively). The inter-assay CV’s were 5.2%, 5.3% and 8%, respectively. EDTA-plasma was used for L-arginine and ADMA determinations, measured by high performance liquid chromatography and precolumn derivatization with *o*-phthaldialdehyde (Sigma Chemicals Co, St.Louis, MO, US) as described in details elsewhere with minor modifications [21]. The inter-assay CV’s were < 5% for both.

### Statistics

For demographic variables proportions or mean values (SD) are given. Other variables are given as mean (SD) or medians (25 and 75 percentiles) as indicated. For group comparisons independen t-test or Mann Whitney U test were used when appropriate. Chi *x*^2^ test was used for comparison of categorial data. Multivariate regression analyses were used to adjust for relevant covariates as appear from Table 1 (i.e. coronary heart disease, heart failure, total cholesterol, fasting glucose, use of medication, creatinine and body mass index).

**Table 1:** Baseline Characteristics of the Study Population According to Groups of Cases (AF) and Controls, Data are Presented as Mean Value (SD) or Proportions

	Cases (AF) (n = 62)	Controls (n = 124)	P-value
Gender (female/male) (n)	18/44	37/87	ns
Coronary heart disease n (%)	21 (34)	17 (14)	0.001
Hypertension n (%)	33 (55)	51 (41)	0.077
Diabetes n (%)	7 (11)	5 (4)	0.058
Heart failure n (%)	9 (15)	1 (0.8)	< 0.001
BMI (kg/m^2^)	25.3 (3.7)	25.5 (3.2)	ns
Creatinine (mmol/L)	84 (22)	81 (24)	ns
Total cholesterol (mmol/L)	5.0 (1.0)	5.5 (1.1)	0.002
HDL-cholesterol (mmol/L)	1.7 (0.6)	1.7 (0.5)	ns
Triglycerides (mmol/L)	1.1 (0.6)	1.1 (0.6)	ns
Fasting glucose (mmol/L)	5.7 (1.7)	5.2 (0.7)	0.006
SBP (mmHg)	142 (21)	147 (17)	ns
DBP (mmHg)	82 (10)	81 (9)	ns
Medications n (%)			
ACE inhibitors	18 (29)	16 (13)	0.007
Beta blockers	24 (39)	20 (16)	0.001
Statins	29 (23)	28 (47)	0.001

SBP: systolic blood pressure; DBP: diastolic blood pressure; LVEF: left ventricular ejection fraction; ACE: Angiotensin converting enzyme. P-values refer to differences between groups.

Skewed data were log transformed before entered in the models. Analyses of trends through quartiles of variables were performed with the Chi *x*^2^ linear-by-linear test for identification of cut-off levels. Logistic regression models were performed to analyse for the associations between the presence of AF and the categorized markers, adjusted for the relevant covariates as described in Table 2. A two-tailed value of P < 0.05 was considered statistically significant. The SPSS for Windows version 15.0 (SPSS Inc, US) was used.

**Table 2:** Levels of Endothelial Cell Markers According to Groups of Cases (AF) and Controls, Data are Presented as Mean Value (SD) if not Otherwise Stated

	Cases (AF) (n = 62)	Controls (n = 124)	P-value Crude	P-value Adjusted^2^
E-selectin (ng/mL)^1^	35.4 (25.8, 48.6)	36.3 (25.8, 48.3)	0.980	
VCAM-1 (ng/mL)^1^	865 (745, 1067)	832 (734, 980)	0.162	
VWF (%)	148 (45)	134 (39)	0.023	0.075
ADMA (umol/L)^3^	0.69 (0.13)	0.62 (0.12)	0.001	0.001
L-arginine (µmol/L)	77 (13)	74 (13)	0.240	
L-arginine/ADMA ratio^3^	114 (23)	123 (27)	0.021	0.037

^1^Median (25, 75 percentiles); Mann Whitney U test; ^2^Adjusted for coronary heart disease, heart failure, total cholesterol, fasting glucose and use of medication; ^3^Additionally adjusted for creatinine and body mass index.

## Results

The number of subjects with blood samples available for this substudy was 62 cases (AF patients) and 124 controls in sinus rhythm. Demographic and comorbidity data of the population according to groups are shown in Table 1. The matching for gender was successful. As can be seen there were significantly higher frequencies in AF-cases compared to controls of coronary heart disease (P = 0.001) and heart failure (P < 0.001). Diabetes and hypertension were also numerically more frequent, although not statistically significant (P = 0.058 and P = 0.077, respectively). Significantly more use of medications like beta-blockers, angiotensin converting enzyme-inhibitors and statins was observed in the AF-group, the latter also indicated by lower levels of total cholesterol in this group (P < 0.002). Furthermore, fasting glucose levels were significantly higher in cases versus controls (P = 0.006).

### Markers of endothelial activation (Table 2)

**Figure 1:**
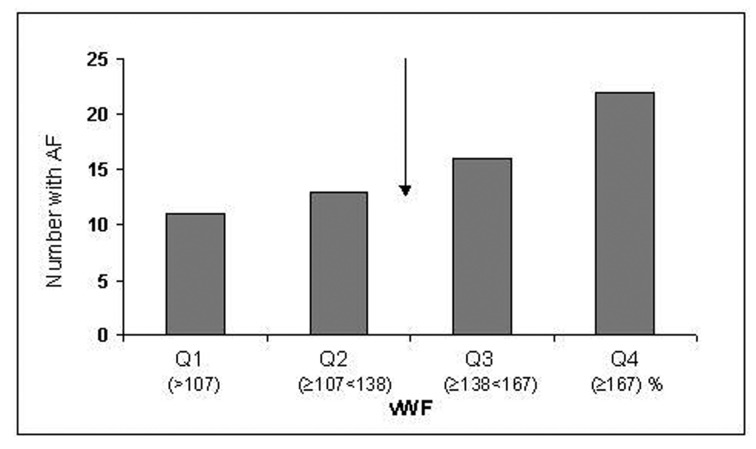
Number of patients with AF according to quartiles of vWF levels. The arrow indicates the cut-off value (median level) used for risk estimation.

There were no significant differences between the AF group and controls in the levels of E-selectin and VCAM-1. Levels of vWF were elevated in AF vs controls (148 (45)% vs 134 (39)%, P = 0.023), however not statistically significant after adjustment for relevant covariates (P = 0.075). When dividing the vWF levels into quartiles there was a non-significant trend for having AF with increasing levels of vWF (P = 0.089). When dichotomizing the levels, there was an increased risk for having AF with levels above median (> 137%), giving an adjusted OR of 1.93 (95%CI 1.03 - 3.58) (P = 0.038) (Fig. 1).

**Figure 2:**
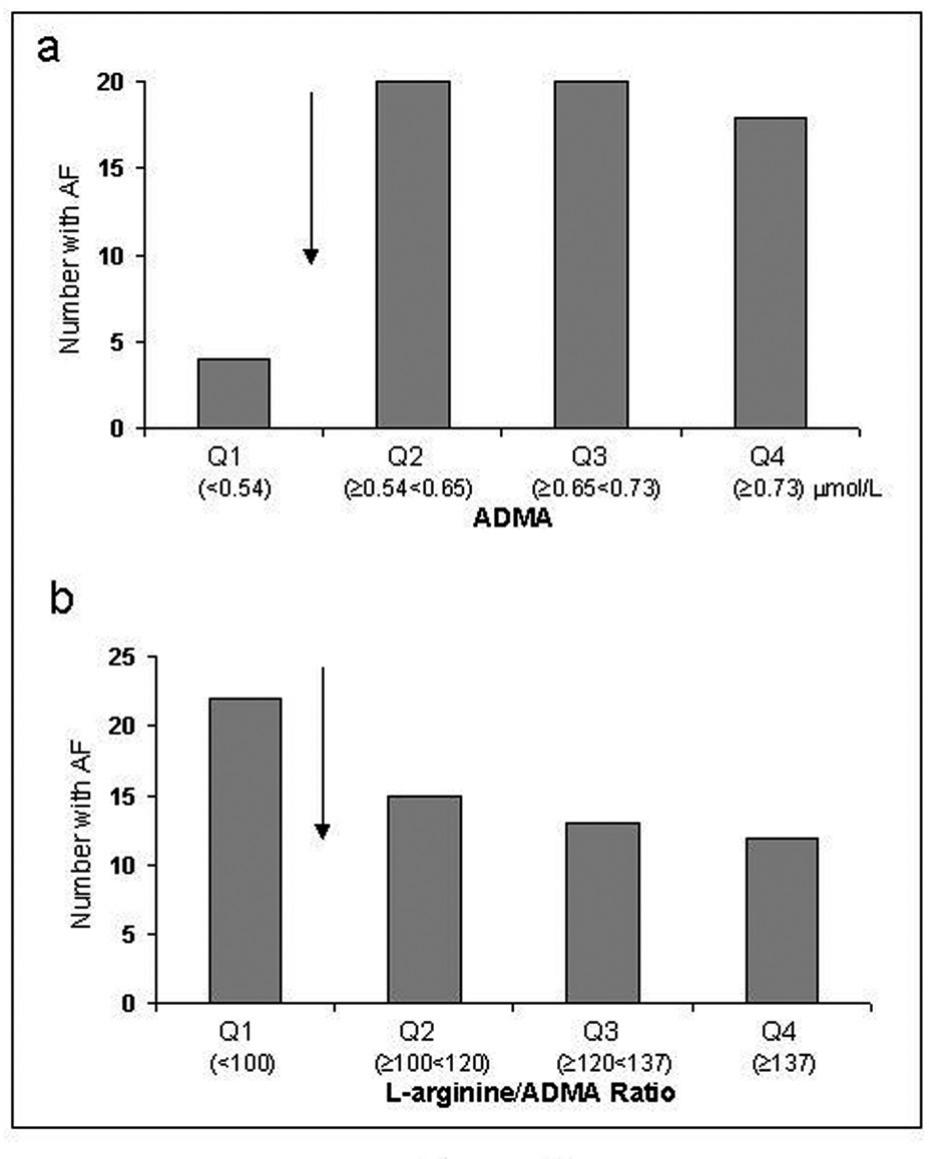
Number of patients with AF according to quartiles of ADMA levels (a) and the L-arginine/ADMA ratio (b). The arrows indicate the cut-off value (25th percentile) used for risk estimation.

Levels of ADMA were significantly elevated in AF vs controls (0.69 (0.13) vs 0.62 (0.12) mmol/L, P < 0.001) and the L-arginine/ADMA ratios were lower (114 (23) vs 124 (27), P = 0.015), still significant after adjustment for covariates (P = 0.001 and P = 0.037, respectively). When dividing the ADMA levels into quartiles there was a significant trend for having AF with increasing levels of ADMA (P = 0.001) with a clear cut-off at the 25th percentile (< 0.54 mmol/L) (Fig. 2a), giving an OR for having AF of 7.16 (95% CI 2.43 - 21.09) (P < 0.001) with higher levels. When adjusted for covariates the OR was 12.46 (95% CI 3.11 - 49.86) (P < 0.001). A similar inverse pattern was seen for the L-arginine/ADMA ratio, with a significant trend for having AF with decreasing levels of the ratio (P = 0.024). With a cut-off value at the 25th percentile (> 100) (Fig. 2b) an adjusted OR for having AF of 0.42 (95%CI 0.17 - 1.01) (P = 0.054), was found.

As gender differences in the risk profile for AF have been shown, differences between the AF and control groups were also analyzed separately in men and women. In men with AF compared to those without, vWF levels were higher (P = 0.028), whereas no differences were observed in women (P = 0.480). ADMA differed significantly in both gender with higher levels in AF patients (P = 0.008 and P = 0.033, respectively).

## Discussion

In this cross sectional study in an elderly population we could demonstrate that the presence of permanent or persistent AF were associated with endothelial dysfunction, assessed by increased levels of vWF, and even more pronounced by the levels of ADMA and the ratio of L-arginine/ADMA.

The relationship between vWF and endothelial dysfunction has been extensively studied, and is thought to be due to release of vWF from the endothelium into the circulation upon endothelial damage or activation [22]. This process seems to be present both in acute and stable conditions [23]. High plasma levels have been reported in several studies on AF, both in comparison with patients in sinus rhythm and also related to AF patients with or without stroke [24]. A linear trend for having AF with increase in vWF levels in women, but not in men was reported in one study [25]. This was not the case in our study, showing a trend only in the total population, and with a significant association between vWF levels and AF only in men.

The mechanisms behind the relationship between vWF and AF have been discussed along with structural abnormalities within the left atrium. Overexpression of vWF was shown in the left atrial appendage in samples from patients with non-valvular AF [26]. It was also reported a strong correlation between abnormal circulating vWF levels and the presence of left atrial thrombus, assessed by spontaneous echo contrast, in patients with non-rheumatic AF [27]. In addition, turbulent flow in AF patients may induce release of vWF from the endothelium in the general circulation. It should, however, be noted that vWF also is produced by megacaryocytes and platelets which may contribute to the circulating levels in pathological states.

The clinical importance of raised vWF levels was investigated among others in the SPAF III study showing higher risk of cardiovascular events, including risk of stroke [28], and very recently it was reported that high plasma vWF levels were an independent risk factor for adverse events in anticoagulated permanent AF patients aged 70 - 80 years [29].

The most striking finding in our study was the significantly higher levels of ADMA, and the lower levels of L-arginine/ADMA ratio in the AF group compared to their gender matched controls. These results are to some degree in line with results from the study by Cengel et al. who mainly focused on differences in ADMA levels in patients with acute vs chronic AF, but where they also found slightly higher levels in chronic AF vs healthy controls with no differences in L-arginine levels [17]. In our population, all patients had chronic AF and they were also older individuals.

The increased levels of ADMA and the impaired L-arginine/ADMA ratio found in AF patients may result in reduction of NO production, which again may contribute to increased risk of thrombosis.

It has been speculated that the irregular, pulsatile blood flow present in AF *per se* may decrease eNOS expression, resulting in reduced NO production, endothelial dysfunction and increased thrombogenicity. Results from animal models have further shown that levels of NO and its bioavalability are reduced also in the atrium during chronic AF, probably due to reduced eNOS activity during turbulent flow [30]. Furthermore, NO has been shown to modulate regulation of ion channels which are involved in electrical remodelling, and AF-induced decrease in NO levels may therefore contribute to maintenance of arrhythmias [31]. Whether these disturbances in NO homeostasis are dependent of or influenced by ADMA and or L-arginine is not fully known. In a study on AF in dogs, increased ADMA levels were found in the atrium in parallel with a reduction in the ADMA degrading enzyme dimethylarginine dimethylaminohydrolase activity-2, and also with increased expression of the protein arginine methyltransferase-1, responsible for the formation of ADMA [32]. These findings indicate that regulation of the L-arginine/ADMA pathway is involved in AF, whether as cause or effect. As “AF begets AF” the endothelial dysfunction that follows AF may in turn contribute to its maintenance.

The increase in ADMA in chronic AF may also be related to the proinflammatory state that is shown to be present in AF as the proinflammatory cytokine tumor necrosis factor-a is known to induce ADMA [33]. Furthermore, ADMA *per se* has been shown to upregulate tissue factor in cell culture studies, indicative of an additional regulatory pathway contributing to thrombogenicity [34].

### Conclusion

Our population of 75-year-old AF patients had significantly impaired endothelial function assessed by increased levels of vWF, and more pronounced by ADMA where a 12-fold increased risk for having AF with higher levels of ADMA was observed.

The results indicate AF in the elderly to be closely associated with the regulatory pathway of NO.
